# Type 1 Immunoglobulin M Cryoglobulinemic Vasculitis in a Patient with Chronic Lymphocytic Leukemia and a History of Hepatitis C Virus: Is There a Link?

**DOI:** 10.7759/cureus.4729

**Published:** 2019-05-23

**Authors:** Naveen Raj

**Affiliations:** 1 Department of Rheumatology, University of Tennessee Medical Center, Knoxville, USA

**Keywords:** cryoglobulin, vasculitis, cll-chronic lymphocytic leukemia, hcv

## Abstract

Hepatitis C virus (HCV) is considered a hepatotropic and, increasingly, a lymphotropic virus. Research suggests an association between HCV infection and the subsequent development of non-Hodgkin lymphomas (NHL). HCV is also a well-known etiologic factor in the development of type II cryoglobulinemic vasculitis while type I cryoglobulinemic vasculitis results from monoclonal immunoglobulin secondary to malignancy. Is there a link among HCV, NHL, and type I cryoglobulinemia? This question is posed in a case of aggressive type 1 cryoglobulinemic vasculitis in a patient with chronic lymphocytic leukemia and a history of HCV. I theorize on an intriguing pathogenesis of how HCV may have led to B cell malignancy and the subsequent development of type I cryoglobulinemic vasculitis in this patient.

## Introduction

Hepatitis C virus (HCV) is a well-known contributor to the development of type II cryoglobulinemic vasculitis, driven by antigen-antibody complexes deposited within small blood vessels. Likewise, type I cryoglobulinemic vasculitis often arises from monoclonal immunoglobulin secondary to malignancy, including chronic lymphocytic leukemia (CLL). This case report details a patient with CLL-associated type I immunoglobulin M (IgM) cryoglobulinemic vasculitis, itself a rare manifestation of CLL. It is also intriguing that this patient had a history of HCV. This raises the possibility of a link among these three conditions, with HCV conferring an increased risk for the development of CLL, which, in turn, led to the development of cryoglobulinemic vasculitis.

## Case presentation

**Figure 1:**
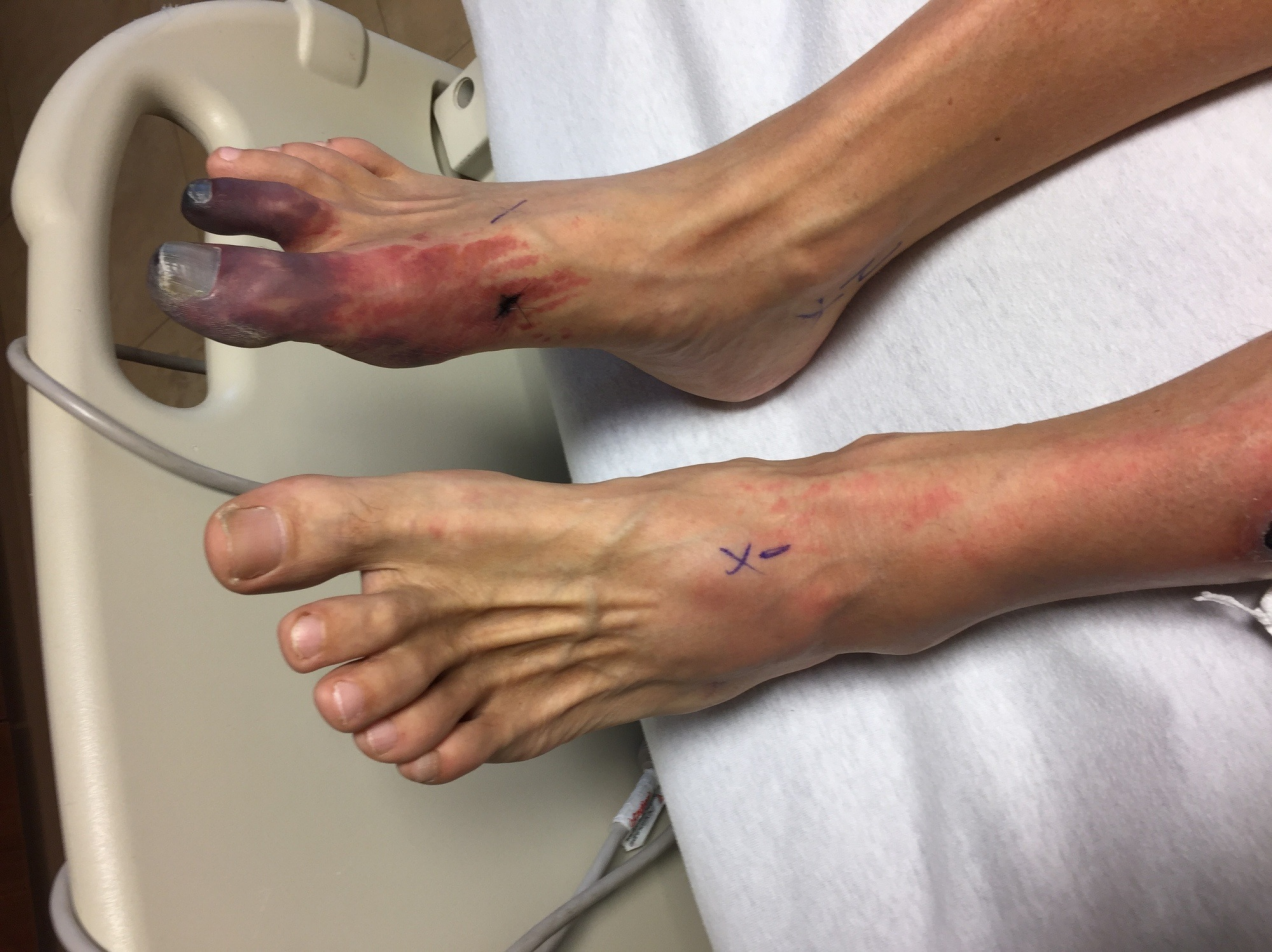
Ischemic lesion to the right foot

A 56-year-old Caucasian man presented to the hospital with a three-day history of a red, painful rash to the first and second digits on the right foot, suggestive of ischemia. The patient stated that three days prior to the development of the rash, he was experiencing numbness and tingling at the site of the eventual lesion. An abdominal aortogram with lower extremity runoff showed patent bilateral renal arteries, patent bilateral common and external iliac arteries, patent right profunda, and three vessel runoff with an intact pedal arch flow. The physical exam was notable for right foot first and second digit duskiness with extreme pain to palpation, along with left foot and leg erythema (Figure 1). Bilateral dorsalis and posterior tibial arteries were palpable. He denied any B symptoms and did not demonstrate any lymphadenopathy on an exam. The rest of the patient’s physical exam was normal. His vital signs were also within the normal range. The patient’s history was notable for CLL diagnosed in 2011 through routine lab work showing leukocytosis, with subsequent confirmation via a bone marrow biopsy. He also had a history of untreated HCV thought to be contracted through his extensive intravenous drug use in the 1990s. He was treated for the HCV in 2012 with ribavirin and beta interferon and was cleared of the virus. As the CLL was asymptomatic and not appearing to progress, no treatment for this was commenced and he was under watchful monitoring by his outpatient oncologist.

**Figure 2:**
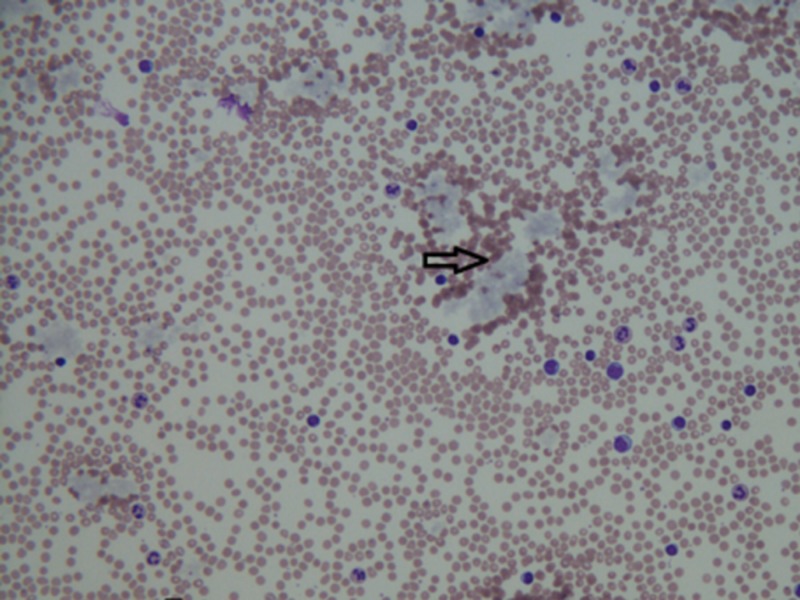
Peripheral blood smear with bluish-gray clumps of cryoglobulins (black arrow)

**Figure 3:**
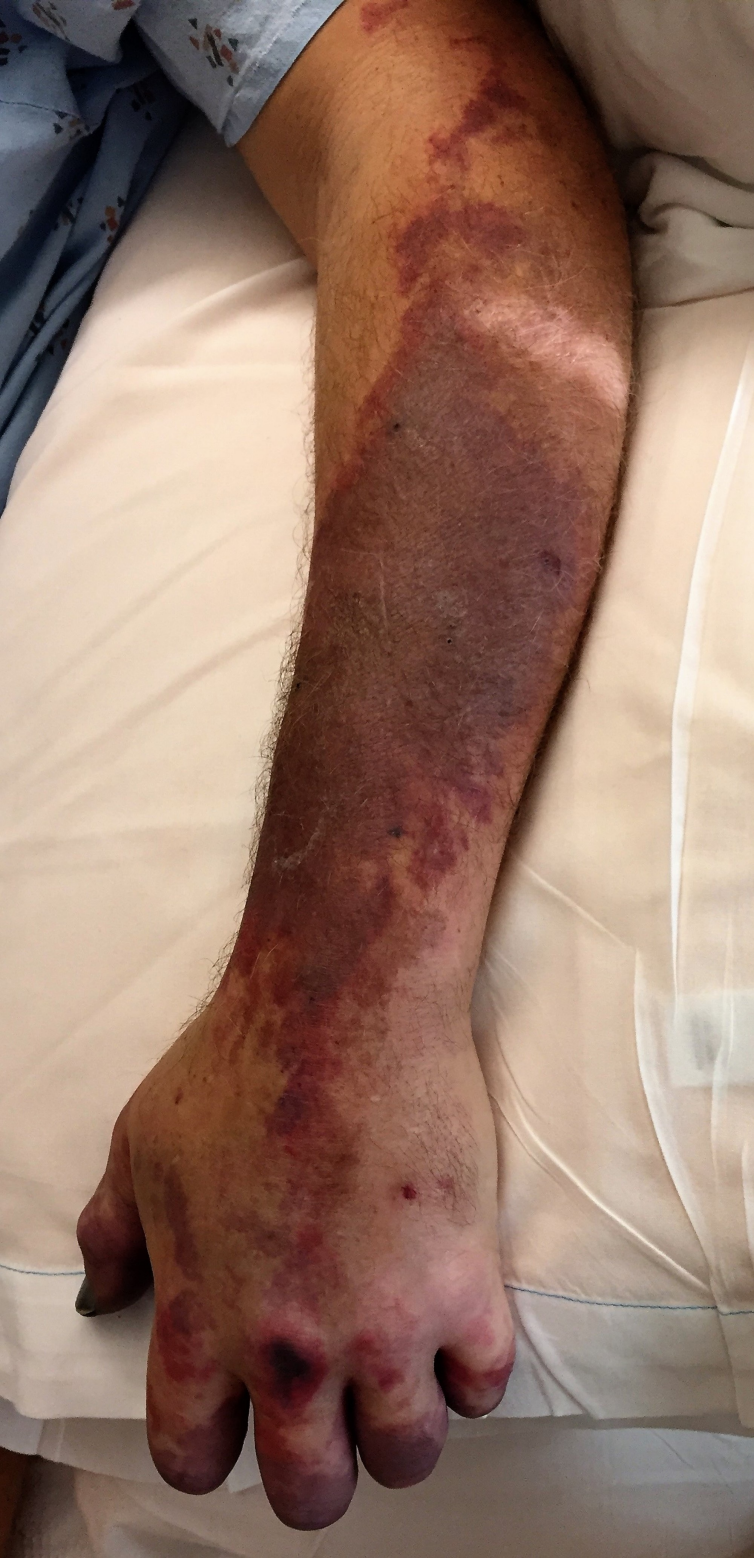
Rapidly progressive ischemia extending over the left forearm

**Figure 4:**
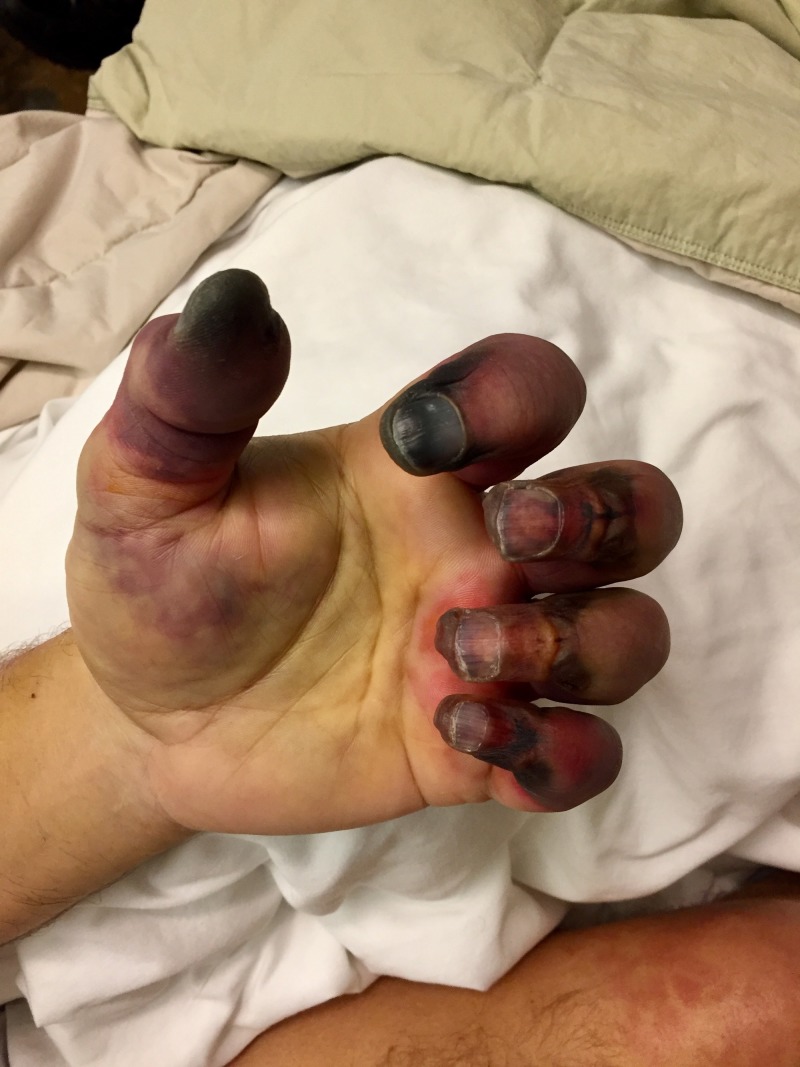
Necrosis of the digits of the left hand, eventually needing amputation

**Figure 5:**
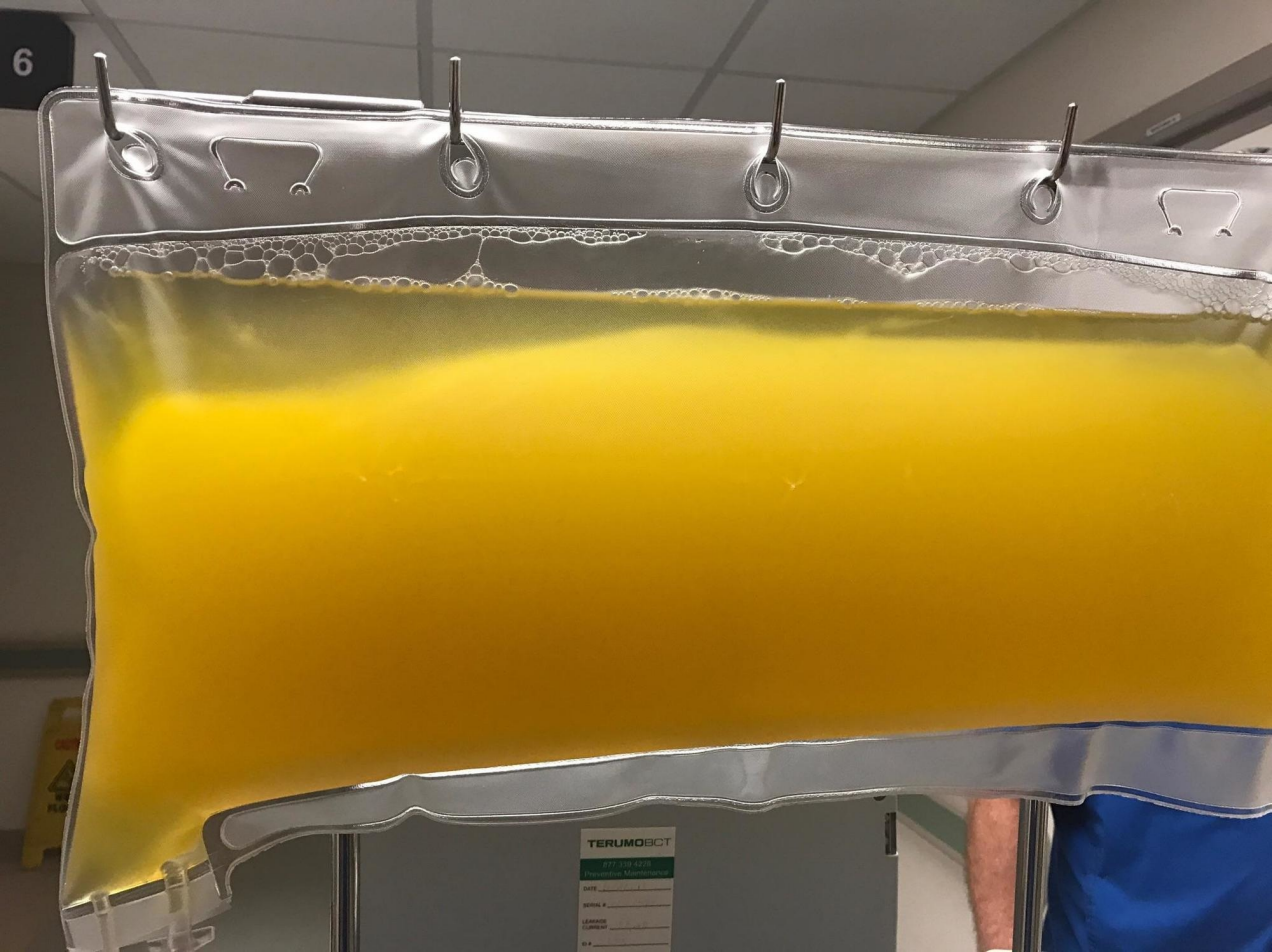
Sediment in collection bag post plasmapheresis

**Figure 6:**
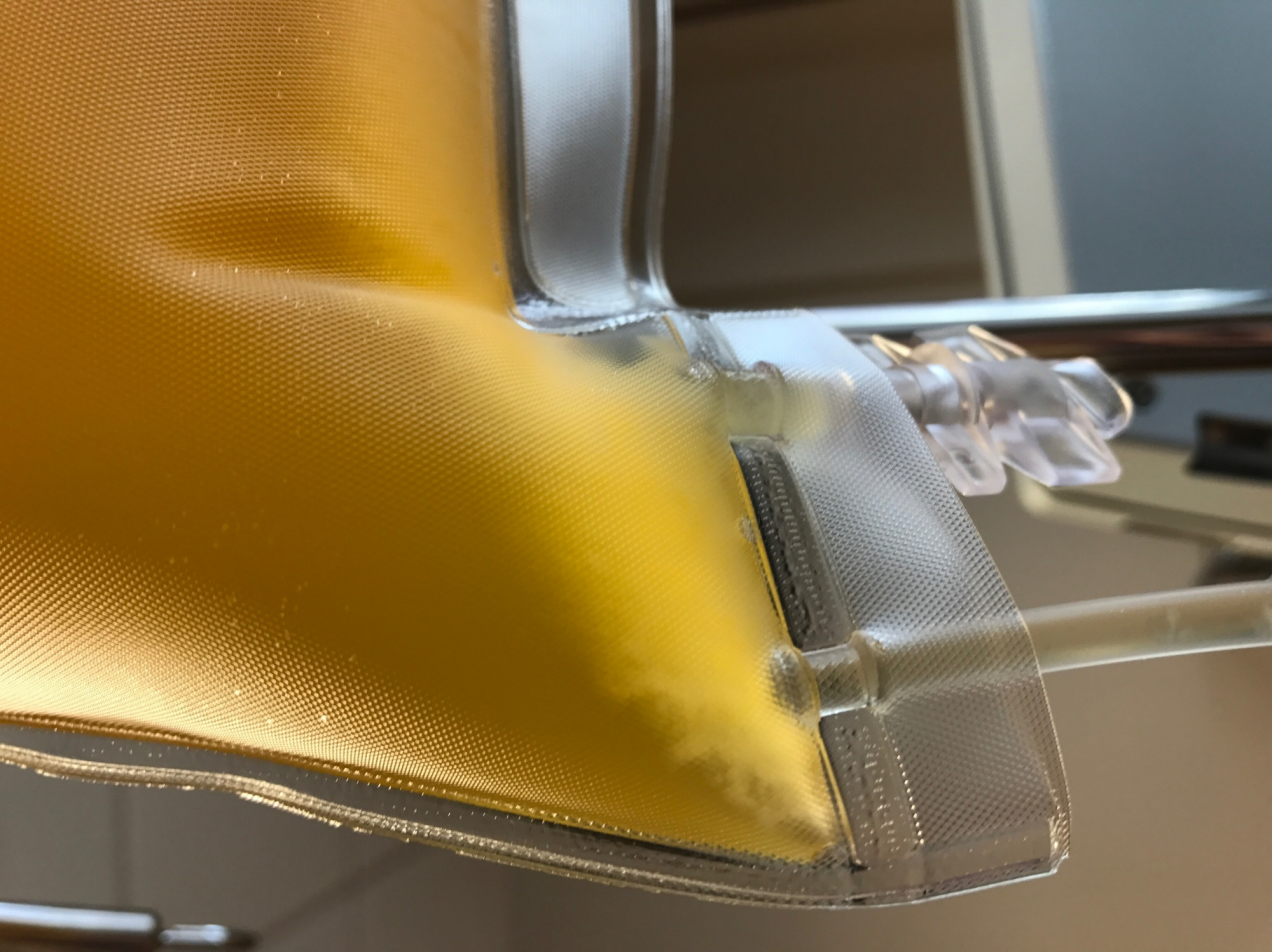
Sediment is thought to be cryoglobulin

As vasculitis was suspected on initial rheumatologic evaluation, the patient was started on prednisone 60 mg daily as workup commenced. A biopsy of the right foot ischemic lesion revealed ischemic necrosis of the epidermis and dermis, with vascular congestion but no evidence of vasculitis and negative direct immunofluorescence. Labs were obtained on admission and during his hospitalization (Tables 1-3). A computerized tomography (CT) scan of his chest, abdomen, and pelvis was negative for malignancy. A transesophageal echocardiogram did not reveal any thrombus. A bone marrow biopsy showed CLL persistence, with an absolute neoplastic lymphocyte count of 19 k/ul. Interestingly, the peripheral blood smears showed an abundance of proteinaceous blue-gray material consistent with cryoglobulins (Figure 2). Four days into the patient’s admission, he developed pain and swelling in his left foot, along with erythema. As the lesions on his left foot continued to worsen, he was treated with a course of high-dose intravenous (IV) methylprednisolone. As it was felt the patient had developed CLL-associated vasculitis, an initial dose of rituximab 375 mg/m^2^ was given according to the CLL dosing guideline. The day after rituximab infusion, bendamustine 100 mg/m^2^ was given over the course of the following two days. Despite these therapies, the patient developed erythema of his left hand and forearm (Figure 3) and his fingers rapidly became necrotic (Figure 4). He also developed ischemia and necrosis of his left upper ear. Due to the aggressive spread of cryoglobulinemic vasculitis, plasmapheresis was commenced on a near-daily basis. Plasmapheresis was striking for the amount of sediment in the collection bag effluent (Figures 5-6). Given the positive cryoglobulin screen, the sediment was thought to be the large quantities of cryoglobulins the patient was producing. Two weeks after his first rituximab dose, he was given a second rituximab dose. He was then discharged home on prednisone 60 mg daily and plans for the continuation of outpatient plasmapheresis and the continuation of combination rituximab and bendamustine treatment. Six days after discharge, he was readmitted for worsening pain and necrosis to his left foot and left hand. The ischemia and necrosis were felt to be secondary to the ongoing cryoglobulinemic vasculitis attack. He was treated with another course of rituximab. Three days later, cyclophosphamide 1000 mg IV was given. The patient continued to deteriorate and he was given rituximab 375mg/m^2^ and cyclophosphamide 1000 mg together 17 days after the initial cyclophosphamide dose. He continued with near-daily plasmapheresis as well as oral steroids during this period. The patient eventually stabilized and did not develop new areas of ischemia. Unfortunately, the areas of prior necrosis were deemed unsalvageable and he subsequently underwent amputation of the distal digits of his left hand with a left forearm fasciotomy, left foot amputation, and amputation of the first two digits of his right foot. The patient was eventually transitioned to oral cyclophosphamide 75 mg daily as well as obinutuzumab (CD-20 directed cytolytic antibody) 1000 mg monthly infusion as the treatment for his CLL. Rituximab was deemed ineffective and discontinued. He also currently undergoes plasmapheresis once a week. He has so far not developed any new necrotic lesions. Aside from the physical exam, trending the Kappa/Lambda (K/L) light chain ratio has been a way of gauging the patient’s response to treatment. As seen in Table 3, the patient’s Lambda light chain number has reduced to a normal range during his treatment course, with subsequent normalization of the K/L ratio. Immunofixation on each cryoglobulin screen showed immunoglobulin M (IgM) monoclonal protein with Lambda light chain specificity. Immunofixation and electrophoresis of the cryoprecipitate revealed type I cryoglobulinemia. The cryoglobulin screen eventually turned negative, as noted in Table 2, attesting to the patient's treatment success thus far. Monitoring CD 19/20 counts is another likely method of assessing treatment efficacy but has, so far, not been pursued by the oncology service. 

**Table 1:** Patient's labs ANA: antinuclear antibody; SPEP: serum protein electrophoresis; Ig: Immunoglobulin; HCV: hepatitis C virus; TB: tuberculosis; ESR: erythrocyte sedimentation rate; CRP: C-reactive protein; RF: rheumatoid factor; CH50: 50% hemolytic complement; ANCA: anti-neutrophil cytoplasmic antibody; WBC: white blood cell; RBC: red blood cell

Value	Result	Reference
ANA	negative	
Urine drug screen	negative	
Lupus anticoagulant	negative	
SPEP	negative	M spike % not observed
Immunoflurescence	normal pattern	
B2 glycoprotein Ab IgG, IgA, IgM	negative	
Lupus anticoagulant Ab, IgG, IgA, IgM	negative	
Hepatitis C Ab	> 11 S/co	S/co ratio negative: 0-0.9, positive: > 0.9
Hepatitis C quantitative	HCV not detected	qualitative assay range 15 IU/mL to 100 million Iu/mL
Hepatitis BS Ag	negative	
Hepatitis BS Ab	negative	
Hepatitis B core Ab	negative	
HIV antigen/antibody	non-reactive	neg <1:32
Cold agglutinin	negative	
Quantiferon TB Gold	negative	
Blood culture	negative	
ESR	4 mm/hr	0-30 mm/hr
CRP	0.5 mg/dL	0-0.8
RF	<10 IU/mL	0-30
Complement C3	56 mg/dL	76-164
Complement C4	< 2 mg/dL	12-48.7
CH50	15 units	42-60
C-ANCA	< 1.20 titer	neg: < 1:20
P_ANCA	< 1.20	neg: < 1:20
Proteinase 3 Ab	< 3.5 U/mL	0-3.5
Myeloperoxidease Ab	< 9 U/mL	0-9.0
Creatinine	1.18 mg/dL	0.76-1.27
Hemoglobin	13.6 G/dL	14-18
WBC	40.6 * 10^3^	4.4-11.0 (10*3)
Urine RBC	negative	negative

**Table 2:** Cryoglobulin screen (%)

1/20/17:	10%
2/27/17:	4%
3/02/17:	13%
3/06/17:	11%
3/29/17:	3%
12/27/17:	Not detected

**Table 3:** Kappa/Lambda light chain ratio, mg/l

Date:	1/20/17	2/14/17	5/17/17	7/28/17	10/2017	Reference:
K:	8.26 K	7.98 K	6.26 K	4.7 K	5.6 K	K: 3.3 - 19.4 mg/L
L:	67.03 L	52.88 L	22.81 L	15.4 L	15.2 L	L: 5.71 - 26.3 mg/L
K/L ratio:	0.12	0.15	0.27	0.31	0.37	K/L ratio: 0.26 - 1.65

## Discussion

Cryoglobulins (CR) are serum immunoglobulins that precipitate at temperatures less than 37° Celsius. They mostly precipitate at 0°-4° Celsius and dissolve when rewarmed [1]. Cryoglobulins are classified into three types. Type I is composed of monoclonal immunoglobulins and usually denotes B cell malignancy. Types II and III are composed of immune complexes (antigen-antibody complexes) and are called mixed cryoglobulinemia. Type II results from the production of a monoclonal rheumatoid factor (RF), such as IgM autoantibody acting against polyclonal IgG class immunoglobulins [2]. Type III consists of polyclonal RF-polyclonal IgG immunoglobulins. Both types II and III indicate immunostimulation and/or infection [1]. Only a monoclonal immunoglobulin is detected in type I cryoglobulinemia. However, in mixed cryoglobulinemia, the RF is complexed with polyclonal immunoglobulins directed against a stimulating agent (viruses, bacteria, or specific immunogens) [1,3]. Cryoglobulinemic vasculitis occurs when cryoglobulins precipitate in the small vessels (venules, capillaries, and arterioles) of different tissues, causing vessel inflammation. The histological hallmark of this condition is leukocytoclastic vasculitis with immune complex deposition on immunofluorescence. Cryoglobulinemic vasculitis can involve many organs, including the skin, peripheral nervous system, and kidneys. HCV is the classic infection that is associated with mixed cryoglobulinemia. It is thought that HCV infects circulating B lymphocytes, stimulating them to ultimately produce monoclonal IgM RF, leading to type II cryoglobulinemic vasculitis. The deposition of CR in and around the blood vessel can lead to ischemia, infarction, and purpura [1]. This patient developed ischemia and purpura of his distal extremities secondary to aggressive cryoglobulinemic vasculitis. What ultimately led to this patient’s cryoglobulinemic vasculitis and its relationship to his prior HCV and CLL is intriguing pathogenesis.

HCV is considered a hepatotropic and possibly lymphotropic virus. While HCV infection frequently leads to chronic hepatitis and is a major cause of hepatocellular cancer, hematological manifestations, such as type II MC or B cell non-Hodgkin lymphoma (NHL), are less common [4]. HCV prevalence in patients with NHL or other chronic lymphoproliferative disorders is between 8% and 32%, depending on the author [5-6]. While a number of epidemiological studies generally support a relationship between chronic HCV infection and an elevated risk for developing NHL, other authors suggest that the association of NHL with HCV infection is related to the geographic distribution of HCV prevalence and to different viral types and subtypes [6].

Morphologically, HCV-associated lymphomas represent a variety of Word Health Organization (WHO) types, including marginal zone, small lymphocytic lymphoma/chronic lymphocytic leukemia, lymphoplasmacytic lymphoma, and diffuse large B cell lymphoma [2]. Three general theories have emerged in an effort to understand an HCV-induced malignancy transformation process: 1) continuous external stimulation of lymphocyte receptors by HCV viral antigens and consecutive B cell proliferation; 2) HCV replication in B cells with resultant oncogenic effects mediated by intracellular viral proteins; 3) permanent B cell damage (mutation of tumor suppressor genes, caused by a transiently intracellular virus - the “hit and run” theory) [4]. More specifically, the theory one holds that the HCV envelope protein E2 binds to the CD81 B cell receptor (BCR). CD81 is known to form co-stimulatory complexes with other BCR’s CD19 and CD21. Stimulation of CD19/CD21/CD81 decreases the B cell activation threshold and may induce B cell proliferation [4,7-8]. Regarding theory two, it is still unclear which signals or signaling pathways mediate HCV-NHL oncogenic transformation. The pro-inflammatory interleukin 6 (IL 6) has a known stimulatory effect on B cells and is thought to contribute to the development of cryoglobulinemia and B-NHL [4,9]. In addition, B-lymphocyte stimulator factor (BLyS) upregulation in HCV-infected B cells can lead to B cell survival and proliferation. Batten et al. demonstrated that transgenic mice overexpressing BLyS develop B cell hyper-proliferation along with the production of high levels of immunoglobulins, RF, and CRs [4,10]. The third concept in HCV-associated B cell malignancy subscribes to the “hit and run” mechanism by which B cells are transformed by transiently intracellular HCV, with no actual intracellular viral replication. Machida et al. found HCV to induce a high mutation frequency of cellular genes in B cells in vitro, suggesting that HCV induces a “mutator phenotype” by causing alterations in proto-oncogenes and tumor suppressor genes, leading to oncogenic transformation of B cells, even though the HCV may have already left the cell [4,11]. Exposure of B cell lines and peripheral B cells to HCV in vitro was associated with a five to 10 fold increase in nucleotide mutations involving the immunoglobulin heavy chain gene, as well as several oncogenic targets. Interestingly, this increased mutation burden was noted in HCV-associated lymphomas and hepatocellular carcinomas (HCC) but was not detected in HCV negative lymphomas or hepatitis B virus-associated HCC [2].

As deduced, the first two theories are dependent on active replicating HCV as the source of B cell proliferation. It then follows that treating and eliminating HCV will remove the oncogenic stimulus. An interesting study by Bumbea et al. aimed to detect immunophenotypic changes of malignant lymphocytes in chronic lymphoproliferative disorders (CLD), particularly CLL, in hepatitis virus infections. The study found a higher frequency of HCV infection in patients with CLD, especially CLL patients. The study also demonstrated a significant change in CLL immunophenotype in patients with viral coinfection, suggesting transformation to a more aggressive disease based on the expression of a lymphoma-like immunophenotype [7]. However, another study of 222 CLL patients by Minuk et al. did not support the hypothesis that HBV or HCV infection play a substantial role in the pathogenesis of CLL and that the results also argued against either virus contributing to a more aggressive course of CLL [12]. Whether this patient’s history of hepatitis C influenced his subsequent development of CLL will not be known, but it is not unreasonable to consider HCV as an etiologic factor. He likely had HCV for some time prior to developing CLL. The CLL, in turn, led to type I cryoglobulinemic vasculitis. Interestingly, the patient’s cryoglobulin screen indicated type I cryoglobulinemia likely from his CLL and not mixed cryoglobulinemia (negative RF) as is usually seen in HCV-associated cryoglobulinemic vasculitis. Perhaps the patient at one time did produce asymptomatic mixed CRs from his untreated HCV, which then resolved upon HCV treatment.

The incidence of CLL-associated cryoglobulinemic vasculitis is quite rare and estimates are hard to determine. Hematologic cancers represent less than a quarter of all cryoglobulinemia cases and type I cryoglobulinemia occurs mainly in B cell malignancies. These include Waldenström macroglobulinemia (WM), multiple myeloma (MM), monoclonal gammopathy of undetermined significance (MGUS), and CLL [13]. In a retrospective study of 64 patients with type I cryoglobulinemia, five (8%) had CLL as an associated disorder. All five patients had the IgG isotype [14]. Skin manifestations are the most common physical finding in type I cryoglobulinemia. These include purpura, livedo reticularis, Raynaud’s phenomenon, acrocyanosis, skin necrosis, ulcers, and infrequently digital gangrene. The cutaneous manifestations are often confined to acral areas (distal limbs, nose, and ears), and can be triggered by cold exposure [13]. Of the 16 patients with necrotic purpura in the Harel et al. study, 14 had an IgG CR whereas only two had an IgM CR like this patient. Cutaneous symptoms were the only manifestation of cryoglobulinemia in 12 patients [14]. This patient, unfortunately, developed severe acral cyanosis, eventually leading to digital gangrene, as the predominant manifestation of his vasculitis. Extracutaneous manifestations of type I cryoglobulinemia include peripheral neuropathy in 19% to 44% of patients, arthralgia in 28%, and renal disease in up to 30% [13]. This patient did experience arthralgias as well as lower extremity peripheral neuropathy but did not develop renal disease.

There are some unusual features of this case. First, the right lower extremity skin biopsy showed a negative immunocomplex mediated process on direct immunofluorescence and no vasculitis. This may be due to an inadequate vessel biopsy sample and only one biopsy was pursued. Interestingly, the peripheral blood smear showed clumps of cryoglobulins and the C4 count was low, indicating complement activation. In addition, while CRP and ESR are often elevated in active vasculitis, they were in a normal range in this patient. The serum protein electrophoresis (SPEP) and serum immunofixation were negative, although the Kappa/Lambda light chain ratio was abnormal, indicating B cell proliferation. There are only a few publications regarding type I cryoglobulinemia treatment. In patients with aggressive/symptomatic disease, treatment strategies targeting the underlying B cell malignancy are employed. This patient had aggressive cryoglobulinemic vasculitis with resulting acral necrosis. His treatment was equally aggressive in an effort to salvage his limbs and included a combination of a number of chemotherapeutic agents, rounds of corticosteroids, as well as ongoing plasmapheresis.

## Conclusions

HCV is a well-known etiologic factor in the development of type II mixed cryoglobulinemia. Recently, evidence has emerged showing HCV to also be lymphotropic, spurring the development of a number of B cell malignancies, including CLL. CLL subsequently is rarely associated with the development of type I cryoglobulinemic vasculitis. A causal link between HCV, CLL, and subsequent type I cryoglobulinemic vasculitis in the same patient is an intriguing etiologic hypothesis that I posit. To my knowledge, there is no previously published case of type I IgM cryoglobulinemic vasculitis in a patient with CLL and a history of hepatitis C. This case also suggests that type 1 and II cryoglobulinemic vasculitis may not be such distinctly separate conditions but rather are of a more fluid nature. While the association could be coincidental, patients with type I cryoglobulinemic vasculitis should be routinely checked for past or current hepatitis C.
